# Unusual Presentation of Maxillary Plasmacytoma in a Young Patient: Diagnostic and Histopathological Insights

**DOI:** 10.1002/ccr3.71426

**Published:** 2025-11-06

**Authors:** Mais Musleh, Suha Aldyat, Waheb Audi, Qossay Alhusein

**Affiliations:** ^1^ Department of Hematology, Faculty of Medicine Al Mouwasat University Hospital Damascus Syria; ^2^ Department of Oral and Maxillofacial Surgery, Faculty of Dentistry Daraa National Hospital Daraa Syria

**Keywords:** jaw, maxillary, plasmacytoma, young

## Abstract

Extramedullary plasmacytoma (EMP) of the maxilla is a rare tumor to be in young patients. Accurate diagnosis requires histopathology, immunohistochemistry, and exclusion of systemic disease. This case highlights the need for early recognition and long‐term surveillance to prevent its progression to multiple myelomas, emphasizing a multidisciplinary approach in managing atypical plasma cell neoplasms.

## 1 Introduction

Plasmacytoma is a rare neoplastic condition arising from monoclonal plasma cells, the terminally differentiated form of B‐lymphocytes [1]. These aberrant plasma cells secrete monoclonal immunoglobulins and may proliferate in osseous or soft tissues, resulting in localized tumor formation [2]. When the disease is restricted to a single site without systemic involvement, it is classified as a solitary plasmacytoma, which may be further categorized into solitary bone plasmacytoma or extramedullary plasmacytoma (EMP) depending on the site of origin [3].

Although the axial skeleton is the most frequent site of involvement, solitary plasmacytomas occurring in the head and neck region—particularly the jaw—are uncommon and often present diagnostic and therapeutic challenges due to their rarity and nonspecific clinical features [4, 5]. Management typically involves local surgical excision and/or radiotherapy, with systemic therapy reserved for cases demonstrating progression toward multiple myelomas [6].

This case report describes an unusual presentation of a solitary plasmacytoma involving the maxillary region in an 18‐year‐old female, who initially presented with localized dental and maxillofacial complaints and was diagnosed with EMP.

## 2 Case Examination

**FIGURE 1:**
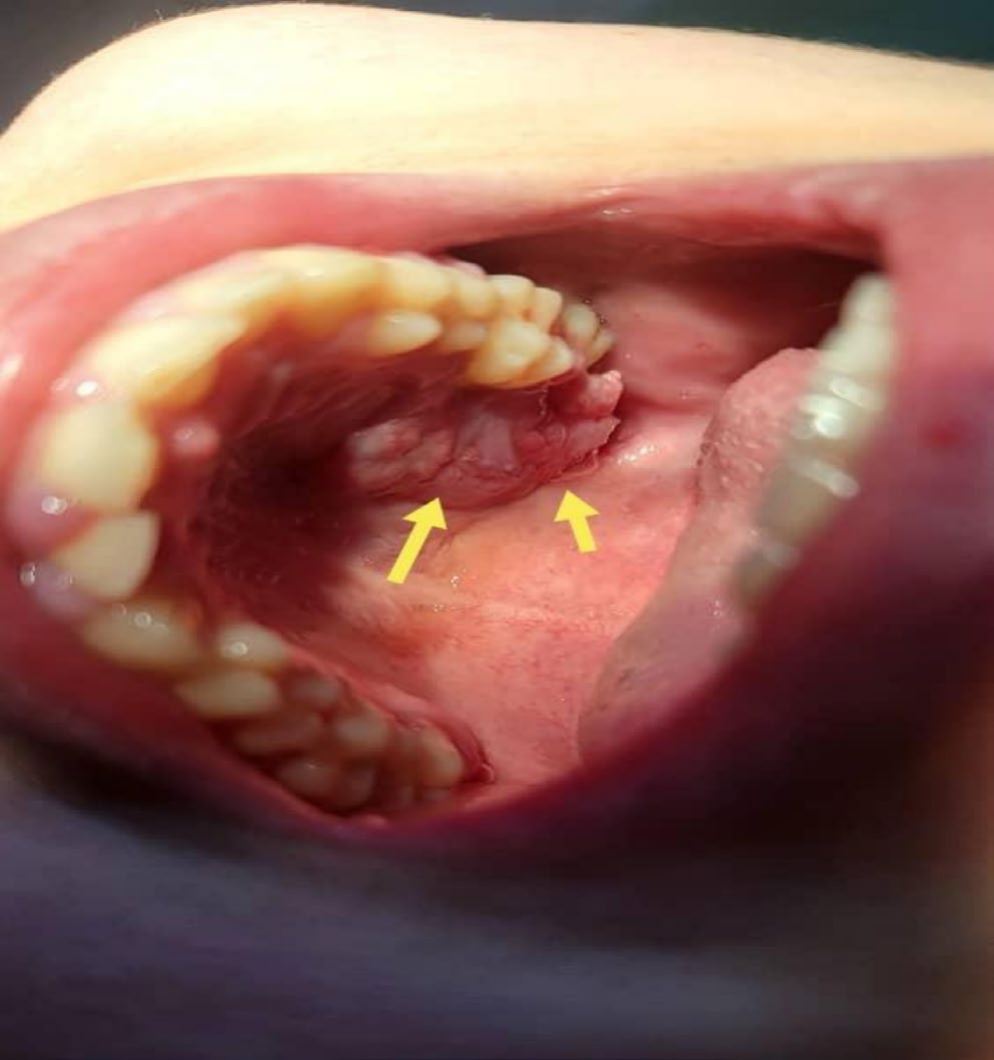
An anatomical view demonstrating the palatal dome and the three alveolar cortices—buccal, palatal, and interradicular—in the upper left maxillary region.

**FIGURE 2:**
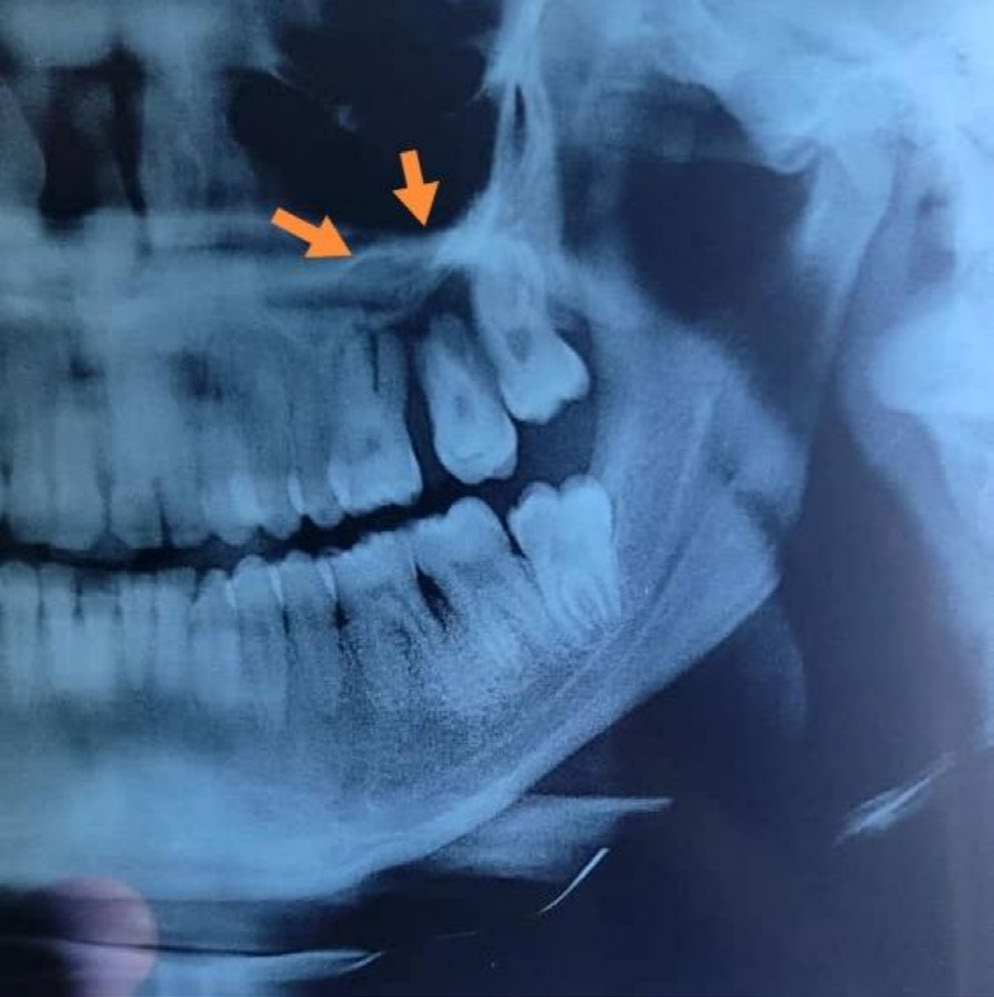
3D volume‐rendered reconstruction, along with intraoral periapical radiography, revealed a well‐defined unilocular radiolucent lesion localized to the region of the left maxillary second molar, measuring approximately 2.2 × 2.3 cm.

An 18‐year‐old female presented to the clinic with complaints of delayed eruption and malalignment of the upper left maxillary molars. Clinical examination revealed no signs of systemic illness. Intraoral assessment demonstrated an intact palatal vault and preservation of the buccal, palatal, and interradicular alveolar cortical plates in the upper left maxilla, as shown in Figure 1. Advanced imaging, including 3D volume‐rendered reconstruction and intraoral periapical radiography, revealed a well‐circumscribed unilocular radiolucent lesion in the region of the left maxillary second molar, measuring approximately 2.2 × 2.3 cm (Figure 2).

## 3 Methods and Differential Diagnosis

**FIGURE 3:**
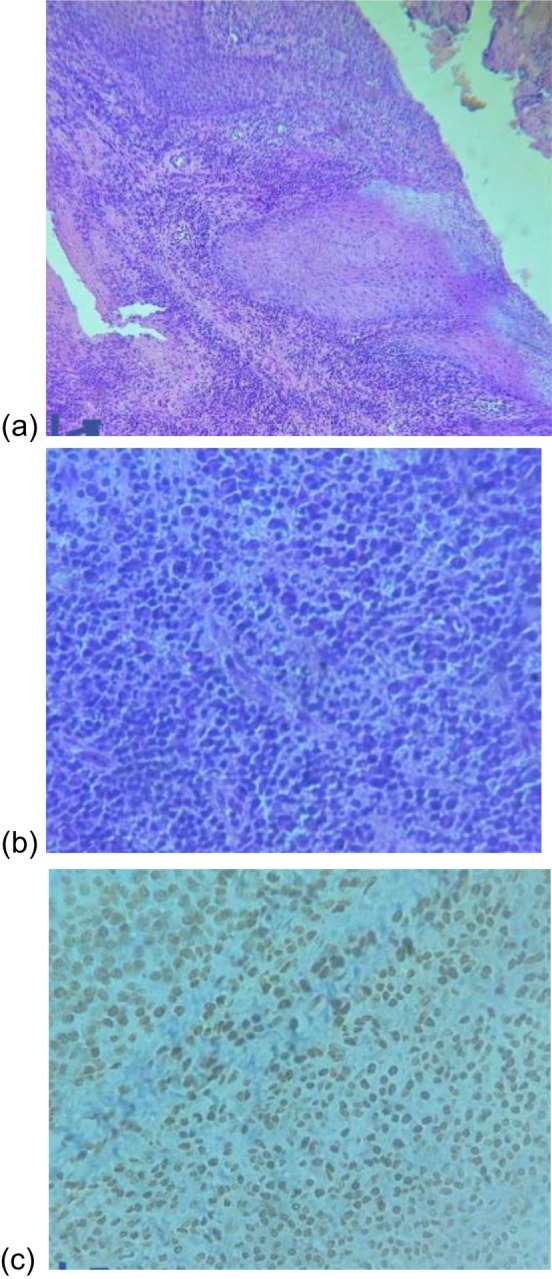
The excised soft tissue specimen measured 25 × 20 × 4 mm. Histological examination revealed a complete replacement of the normal tissue architecture beneath the overlying oral mucosa by markedly inflamed connective tissue (a). The inflammatory infiltrate consisted predominantly of pleomorphic white blood cells with evident mitotic activity, with plasma cells being the dominant population (b). Immunohistochemical staining for CD138 demonstrated strong positivity, confirming the presence of a dense population of monoclonal plasma cells (c).

An excisional biopsy of the associated soft tissue was performed. Histopathological examination demonstrated markedly inflamed connective tissue with near‐total architectural replacement (Figure 3). The tissue was infiltrated by pleomorphic white blood cells exhibiting increased mitotic activity. The predominant cell population consisted of plasma cells. Immunohistochemical staining confirmed strong CD138 positivity, kappa few positive cells, supporting the diagnosis of a monoclonal plasma cell proliferation.

A comprehensive laboratory workup was conducted. Results were within normal limits, including a white blood cell count of 8800/μL, hemoglobin level of 12.4 g/dL, platelet count of 309 × 10^9^/L, erythrocyte sedimentation rate (ESR) of 12 mm/h, and serum creatinine of 0.45 mg/dL. Serum calcium was 9.4 mg/dL, ruling out hypercalcemia. Serum protein electrophoresis revealed no monoclonal spike, and urine protein electrophoresis was negative for Bence Jones proteins. Serum immunoglobulin analysis—including both heavy and light chains—was within normal reference ranges. Beta‐2 microglobulin (β2M) levels were also normal.

Bone marrow biopsy showed normocellular marrow (approximately 65%) without morphologic evidence of plasma cell infiltration. However, flow cytometry identified a small plasma cell population (6%) with corresponding CD138 expression, kappa light chain 8%. Cytogenetic analysis demonstrated a normal female karyotype (46,XX). Whole‐body PET‐CT imaging revealed no evidence of systemic disease or additional extramedullary lesions.

## 4 Conclusion and Result

Based on the clinical, radiological, histopathological, and immunophenotypic findings, a diagnosis of EMP was established. Given the absence of systemic involvement or diagnostic criteria for multiple myelomas, the patient was placed on a structured intensive follow‐up protocol. This includes periodic laboratory testing and radiographic imaging to monitor for recurrence and ensure early identification of any progression toward systemic disease.

## 5 Discussion

EMP is a rare manifestation of plasma cell neoplasms, accounting for approximately 3% of all plasma cell dyscrasia. Unlike solitary bone plasmacytoma, which arises within the bone marrow, EMP originates in soft tissues without direct bone marrow involvement [1, 2]. EMPs most commonly affect the upper aerodigestive tract—including the nasal cavity, paranasal sinuses, oropharynx, and larynx—due to the abundance of lymphoid tissue in these regions [3]. Involvement of the oral cavity or maxillary soft tissues is particularly rare, especially in pediatric and adolescent populations.

EMP tends to affect males more frequently, with a male‐to‐female ratio of approximately 3:1, and typically presents in the fifth to seventh decades of life [5]. The occurrence of EMP in an 18‐year‐old female, as presented in this case, is therefore highly unusual.

The diagnostic workup for EMP requires exclusion of systemic plasma cell disorders such as multiple myelomas. Diagnostic criteria, as outlined by the International Myeloma Working Group (IMWG), include histopathological confirmation of monoclonal plasma cell infiltration in extramedullary soft tissue; absence of clonal plasma cell proliferation in the bone marrow or < 10% plasma cells, with no evidence of systemic myeloma–defining events (CRAB criteria); normal skeletal survey or PET‐CT scan with no evidence of osteolytic lesions; no or minimal serum/urine monoclonal protein; and normal renal function, calcium levels, and complete blood count [7, 8].

In this case, the patient presented with a localized lesion in the left maxillary region. Imaging studies revealed a unilocular radiolucent lesion measuring 2.2 × 2.3 cm, without evidence of osseous destruction elsewhere. Histopathology demonstrated a dense infiltrate of atypical plasma cells with marked pleomorphism and mitotic activity. Immunohistochemistry confirmed CD138 positivity, consistent with a plasma cell phenotype.

Importantly, the patient's laboratory evaluation—including complete blood count, renal function, calcium level, and serum/urine protein electrophoresis—was within normal limits. No Bence Jones protein was detected, and PET‐CT imaging revealed no systemic disease. The absence of systemic involvement, combined with the histological and immunophenotypic features of the lesion, supports the diagnosis of an EMP, as low‐level plasma cell presence can occur in otherwise healthy individuals or as a reactive process.

The management of EMP generally involves localized therapy, with treatment selection determined by lesion site, resectability, and anticipated impact on function and cosmesis. In our case, the patient presented with a well‐circumscribed, localized maxillary lesion, which was completely resectable [9]. Surgical excision was performed, and histopathology confirmed negative margins with no residual neoplastic plasma cells, indicating complete removal.

Although radiotherapy (40–50 Gy over 4–5 weeks) is considered the standard of care for EMP—especially for unresectabl [10] incompletely excised, or functionally sensitive lesions—it was not administered in our case due to th combination of complete surgical excision, absence of systemic involvement, and limited availability of radiotherapy at our center. Literature also suggests that outcomes for extramedullary lesions may differ from bony solitary plasmacytomas, with extramedullary disease demonstrating a more favorable prognosis and higher local control rates [11].

Systemic chemotherapy is generally reserved for cases demonstrating progression to multiple myelomas or for patients with high‐risk features [12, 13]. Given the patient's young age, absence of systemic disease on PET‐CT, normal bone marrow cellularity with minimal plasma cell infiltration (6% by flow cytometry), and the lack of monoclonal proteinemia, systemic therapy was deemed unnecessary.

The patient was referred for long‐term follow‐up under hematology and maxillofacial oncology services. Follow‐up plans include periodic clinical evaluations, laboratory assessments (including serum protein electrophoresis and free light chain assays), and imaging as indicated to monitor for local recurrence or progression to multiple myelomas.

In conclusion, EMP of the maxilla is an uncommon plasma cell neoplasm, especially in adolescents. In this case, an 18‐year‐old female presented with a localized maxillary lesion, diagnosed as EMP based on histopathological features and strong CD138 immunopositivity. Systemic workup—including serum and urine protein electrophoresis, immunoglobulin studies, bone marrow biopsy, and PET‐CT—revealed no evidence of multiple myelomas.

Given the potential for disease recurrence or progression, particularly in young patients, long‐term follow‐up with periodic laboratory testing and imaging is essential. This case reinforces the importance of early recognition, complete diagnostic assessment, and vigilant monitoring in rare presentations of EMP.

## Author Contributions

**Mais Musleh:** conceptualization, formal analysis, methodology, resources, software, supervision, visualization, writing – original draft, writing – review and editing. **Suha Aldyat:** conceptualization, data curation, resources, validation, writing – original draft. **Waheb Audi:** funding acquisition, investigation, resources. **Qossay Alhusein:** conceptualization, data curation, supervision, validation.

## Disclosure

Qossay Alhusein is the guarantor of this work.

## Ethics Statement

The authors have nothing to report.

## Consent

Written informed consent was obtained from the patient for publishing this case report and any accompanying images.

## Conflicts of Interest

The authors declare no conflicts of interest.

## Data Availability

All data (of the patient) generated during this study is included in this published article and its supporting information files.
